# Colon-Targeted Mucoadhesive PLGA Microspheres Loaded with *Ramulus Mori* Alkaloids for Enhanced Water-Soluble Drug Delivery in Ulcerative Colitis Treatment

**DOI:** 10.3390/molecules30091878

**Published:** 2025-04-23

**Authors:** Mo Wang, Yu Jiang, Zhiyang Chen, Dengbao Jiang, Xuan Jiang, Jun Ye, Hongliang Wang, Yuling Liu

**Affiliations:** 1 State Key Laboratory of Bioactive Substance and Function of Natural Medicines, Institute of Materia Medica, Chinese Academy of Medical Sciences & Peking Union Medical College, Beijing 100050, China; wangmo@imm.ac.cn (M.W.); 17865562120@163.com (Y.J.); chenzhiyang@imm.ac.cn (Z.C.); jdbao2000@163.com (D.J.); jxuan635@163.com (X.J.); yelinghao@imm.ac.cn (J.Y.); 2 Beijing Key Laboratory of Drug Delivery Technology and Novel Formulation, Institute of Materia Medica, Chinese Academy of Medical Sciences & Peking Union Medical College, Beijing 100050, China; 3 School of Traditional Chinese Medicine, Shenyang Pharmaceutical University, Shenyang 110016, China; 4 University-Town Hospital of Chongqing Medical University, Chongqing 401331, China

**Keywords:** ulcerative colitis, *Ramulus Mori* alkaloids, microsphere, anti-inflammatory, PLGA

## Abstract

Ulcerative colitis (UC) is a chronic inflammation disease with severe impact on quality of life, with limited treatment options. *Ramulus Mori* alkaloids (SZ-A) from Morus alba show promise for UC treatment due to their safety and pharmacological effects, including anti-inflammation and barrier repair. However, their clinical use has been limited by gastrointestinal flatulence as a side effect due to their pharmacological action as an α-glucosidase inhibitor targeting the small intestine following oral administration. Therefore, constructing a colon-targeted formulation to deliver SZ-A is an advantageous strategy to improve UC therapy. In this study, we used the complex formed by thiolated hyaluronic acid, which has mucosal adhesion and inflammation-targeting properties, and SZ-A as an intermediate carrier and prepared sodium alginate-modified PLGA microspheres (SZ-A@MSs) with the double emulsion method to achieve efficient encapsulation of SZ-A. Specifically, sodium alginate serves as a gastric acid protectant and microbiota-responsive material, enabling the precise and responsive release of microspheres in the colonic region. SZ-A@MSs have a particle size of about 30 µm, a drug loading of about 12.0%, and an encapsulation efficiency of about 31.7% and function through intestinal adhesion to and targeting of inflammatory sites. SZ-A@MSs showed antioxidant and anti-inflammatory abilities in Raw264.7 cells. In vivo imaging results suggest that SZ-A@MSs have good colon site retention and sustained-release effect. Pharmacodynamic results show that SZ-A@MSs display good efficacy, including the ability to inhibit weight loss, inhibit colonic atrophy, and inhibit the secretion of inflammatory factors. In conclusion, SZ-A@MSs have good colon-targeting properties, can improve therapeutic effects, and provide a potential treatment method for UC.

## 1. Introduction

Ulcerative colitis (UC) is a chronic non-specific inflammatory bowel disease with an incompletely understood etiology, primarily affecting the mucosa and submucosa of the colon and rectum [1]. The disease course is protracted, often characterized by alternating episodes of relapse and remission, severely affecting the quality of life of patients and imposing a significant medical burden on society [2]. Currently, there is a wide variety of clinical therapeutic drugs for UC, which can be broadly categorized into aminosalicylates, corticosteroids, immunomodulators, biologics, and small-molecule drugs based on their mechanism and clinical applications [3]. Despite the availability of numerous treatment options, the cure rate for UC patients is still far from ideal, with only about 40% of patients being cured [4]. Existing drugs have high side effects with long-term use; thus, there is an urgent need for the development of drugs with good efficacy and high safety.

In recent years, natural small-molecule compounds have attracted much attention in the field of UC therapy due to their advantages of safety and low toxicity, as well as multiple biological activities [5]. *Ramulus Mori* alkaloids (SZ-A), a total alkaloid extract of mulberry branches, is a plant-derived natural drug approved by the NMPA in 2020 for the treatment of type 2 diabetes [6]. Oral administration of SZ-A can also significantly alleviates DSS-induced colitis in mice by improving the intestinal barrier, reducing serum levels of oxidative stress and pro-inflammatory factors, regulating the intestinal flora, and restoring the Th17/Treg balance. However, due to its function as an α-glucosidase inhibitor, oral administration of SZ-A leads to side effects such as gastrointestinal flatulence [7]. To reduce the side effects of oral administration of SZ-A at the small intestinal site, it is necessary to design a colon-targeted delivery system to reduce the release of SZ-A at the small intestinal site. Currently, many plant-derived drugs use colon-targeted delivery systems. Baicalin was successfully prepared as colon-targeted particles with pH-sensitive properties by coating with Eudragit S100, which has good colon-targeting properties to enhance therapeutic efficacy [8]. Gingerol oil colon-targeted pellets were prepared with the extrusion/spherization method to provide reliable experimental support for therapeutic selection of UC [9].

As a commonly used dosage form for colon-specific release, colon-targeted microspheres generally suffer from low encapsulation efficiency and significant burst release when encapsulating water-soluble drugs [10]. Particularly during the preparation process using the emulsion solvent evaporation method, drugs tend to migrate from the organic phase to the aqueous phase or diffuse from the inner aqueous phase to the outer aqueous phase, making it difficult to achieve effective encapsulation. To address these limitations, researchers have developed strategies including hydrophobic prodrug modifications to reduce drug hydrophilicity, composite carrier systems incorporating chitosan or alginate to enhance drug/carrier interactions, surface modification with PEG to optimize microsphere properties [11], multi-layer coating technologies to delay drug release [12], microfluidic technologies to precisely control microsphere size and drug distribution [13], and process parameter optimization through adjustments in emulsification speed, temperature, and solvent removal rates [14]. However, the drug-loading capacity and encapsulation efficiency of microspheres remain suboptimal and further research and refinement are required to overcome these limitations.

Poly(lactic-co-glycolic acid) (PLGA) is a biodegradable functional polymer organic compound with good biocompatibility, non-toxicity, and excellent film-forming and encapsulation properties [15]. PLGA can serve as a carrier for SZ-A, and microspheres prepared with the double emulsion method can isolate the drug inside from the external environment, enabling long-term sustained-release delivery of the drug [16]. However, the hydrophilic nature and low molecular weight of SZ-A result in suboptimal encapsulation efficiency due to drug leakage through microsphere surface pores and internal channels during preparation. To overcome this challenge, thiolated hyaluronic acid (HA-SH) was incorporated into the internal aqueous phase to enhance phase stability and improve SZ-A encapsulation efficiency while imparting mucoadhesive properties to the microspheres [17,18]. However, since an acidic environment accelerates the degradation and release of PLGA microspheres, it is necessary to modify the microspheres [19]. Sodium alginate-modified microspheres can enhance both gastric acid resistance and colon-targeting capability of PLGA microspheres [20]. As a natural polysaccharide, alginate has excellent biocompatibility, biodegradability, and pH-sensitive properties, making it a preferred carrier for colon-targeted delivery [21]. This modification protects the microspheres from premature degradation in gastric acid and shields the drug from premature exposure, thereby ensuring precise drug release at specific sites in the colon. The combination of PLGA and sodium alginate achieves high efficacy and safety in colon-targeted drug delivery through a time-environment double trigger mechanism and multi-layer structural design. PLGA acts as a water-soluble drug carrier and sodium alginate protects the microspheres and ensures their precise positioning in the colon.

This study developed sodium alginate-modified PLGA microspheres via a double emulsion method, utilizing HA-SH/SZ-A complex as an intermediate carrier (SZ-A@MSs), to achieve precise colon-targeted delivery of SZ-A. The SZ-A@MSs adhere to the colonic mucosa and sustain the release of SZ-A, exerting localized anti-inflammatory, immunomodulatory, and intestinal mucosal protective effects in the colon. The development of pH-sensitive and colon mucosa-adhesive colon-targeted microspheres establishes a multi-level synergistic intelligent targeting strategy for UC therapy, enabling efficient site-specific delivery of water-soluble natural component-based drugs.

## 2. Results and Discussion

### 2.1. Preparation and Characterization of SZ-A@MSs

**Figure 1:**
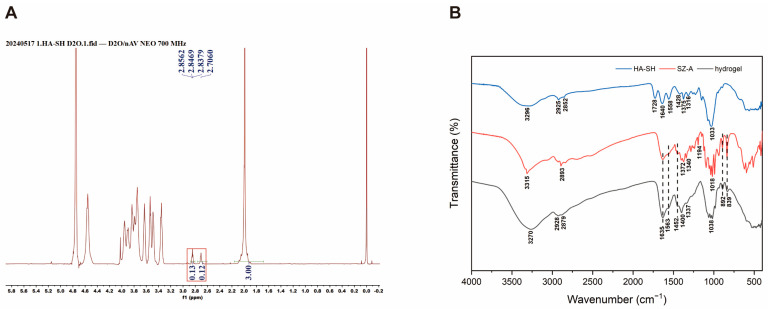
(**A**) NMR hydrogen spectrum of HA-SH. (**B**) Infrared spectrogram of the gel.

Based on the property of sulfhydryl groups to confer mucoadhesion to polysaccharides, the sulfhydryl groups were linked to hyaluronic acid [22]. The successful synthesis of thiolated-HA polymer HA-SH was demonstrated by 1H NMR with a calculated degree of substitution of 6%. (Figure 1A). Mixing SZ-A with synthesized HA-SH can spontaneously form a hydrogel. To demonstrate and explain the gelation mechanism, the hydrogel was measured by FTIR (Figure 1B). From the infrared spectrum of the gel, it can be seen that the absorption peaks of the hydrogel basically include the characteristic group peaks of HA-SH and SZ-A. At 3270 cm^−1^, the hydrogel showed a distinct absorption peak and the position of the peak decreased, accompanied by broadening and intensity enhancement compared to HA-SH and SZ-A. These spectral modifications are characteristic of hydrogen bonding between N-H and O-H groups. This indicates that the hydrogel formed by HA-SH and SZ-A is due to hydrogen bonding between them.

**Figure 2:**
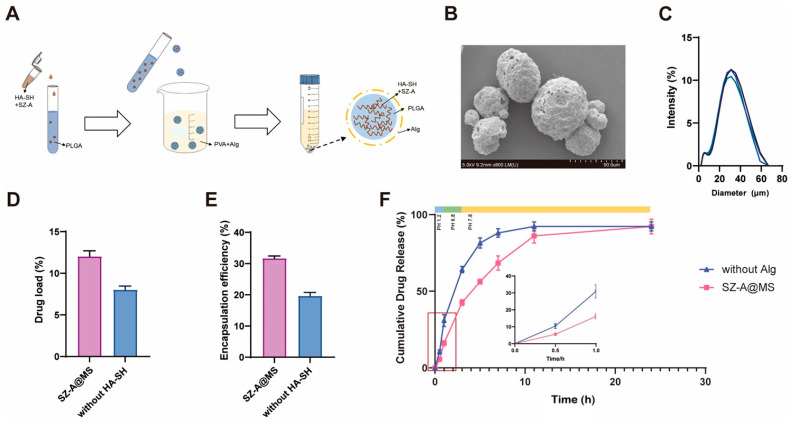
Preparation and characterization of SZ-A@MSs. (**A**) Schematic diagram of the SZA-SMs. (**B**) Particle size. (**C**) Scanning electron microscope (SEM). (**D**) Drug load. (**E**) Encapsulation efficiency. (**F**) Cumulative drug release in vitro. The red rectangular indicates the magnified region (0–1 h). Data are presented as the mean ± SEM. Alg, sodium alginate.

The microspheres were prepared according to the following steps (Figure 2A). The microspheres appeared spherical under SEM (Figure 2B). The porosity of the microspheres was 9.11% ± 1.27%. The particle size of the SZ-A@MSs, measured using a Malvern laser particle-size analyzer, was approximately 30 μm (Figure 2C). The SZ-A content in the microspheres was measured by HPLC, wherein SZ-A@MSs without the HA-SH group had no HA-SH added to the inner aqueous phase. The results showed that the addition of HA-SH to the inner aqueous phase can significantly improve the drug loading and encapsulation efficiency. The drug loading of SZ-A@MSs was approximately 12.0% (Figure 2D), and the encapsulation efficiency was approximately 31.7% (Figure 2E). The mixture of HA-SH and SZ-A has the property of forming hydrogels, which can significantly improve the stability of primary emulsion (W1/O) through hydrogen bonding and reduce the leakage of water-soluble drugs during the preparation process [23].

The microspheres were placed in different pH environments to simulate in vitro gastric fluid (pH 1.2), small intestinal fluid (pH 6.8), and colonic fluid (pH 7.8) (Figure 2F). Among them, SZ-A@MSs without the Alg group represent microspheres without sodium alginate modification. The results showed that all microspheres exhibited a slow release state in accordance with the first-order drug release model, and the sodium alginate-modified microspheres, SZ-A@MSs, were significantly better at slowing down the release of the microspheres. The strategy of surface modification of PLGA microspheres with sodium alginate effectively solved the instability problem of traditional PLGA microspheres in the gastric acid environment [24]. As a pH-sensitive natural polysaccharide [25], sodium alginate can significantly reduce the destructive effect of gastric acid on PLGA, thereby protecting the premature release of drugs in the stomach and small intestine [20]. When the microspheres reach the colon, the gel layer gradually swells and disintegrates, triggering the sustained-release behavior of the drug from the PLGA microspheres, and sodium alginate has a microbiota-triggered effect [26].

**Figure 3:**
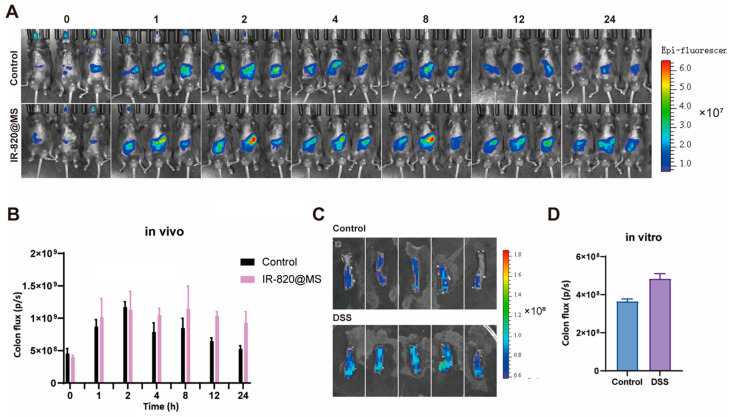
(**A**,**B**) Results of the in vivo imaging system of IR-820@MSs in colitis mice. (**C**,**D**) Comparison of IR-820@MSs adhesion to isolated colonic sites in normal and colitis mice. Data are presented as the mean ± SEM.

Microspheres containing fluorescent dyes (IR-820@MS) were prepared and administered orally to DSS-induced model mice. DSS-induced model mice can better simulate the colon condition of diseased mice. Mice in the control group received the same amount of IR820 fluorescent dye solution orally (Figure 3A,B). Through fluorescence imaging results and quantification of the relative fluorescence intensity of each mouse, the results showed that compared with the control group, stronger fluorescence signals were observed at the 12 h and 24 h time points, and the fluorescence signal attenuation amplitude was smaller, indicating that the fluorescent microspheres stayed in the intestinal tract of the mice for a longer time, proving that the microspheres have a good colon-targeting effect, which can prolong the retention time of the drug in the body to target colon tissue.

To evaluate the adhesion properties of the microspheres, colons from healthy mice and DSS-induced colitis mice were obtained and incubated with IR820 fluorescent microspheres in vitro at 37 °C for 30 min with shaking and then washed with PBS. Fluorescence imaging results showed that there was still a certain amount of microsphere residue in the colon tissue after repeated washing, indicating that the microspheres had a certain degree of adhesion (Figure 3C,D). Compared to the control group, more fluorescent microspheres remained in the colon tissue of the DSS group, indicating that the microspheres adhered more to the inflammation site and that the microspheres had an inflammation-targeting effect. In vitro adhesion experiments showed that the microspheres had the effect of mucosal adhesion and inflammation targeting. This is because the introduction of sulfhydryl groups (-SH) endows HA-SH with mucosal adhesion ability, which can reversibly bind to cysteine-rich proteins in the colonic mucus layer to form disulfide bonds, thereby prolonging the residence time of the microspheres on the colonic epithelium [27]. And HA-SH has abundant carboxyl groups, which helps it to remain at the positively charged inflammatory site [28]. Sites of inflammation in the colonic mucosa trigger the accumulation of positively charged proteins, including transferrin, bactericidal/permeability-increasing proteins, and antimicrobial peptides [29]. At the same time, hyaluronic acid, as a natural ligand of the CD44 receptor, can actively target the highly expressed CD44 receptor at the inflammatory colonic site, thereby achieving drug accumulation in the lesion area [30].

### 2.2. Mesoscopic Simulations of SZ-A@MSs Based on Dissipative Particle Dynamics (DPD)

**Figure 4:**
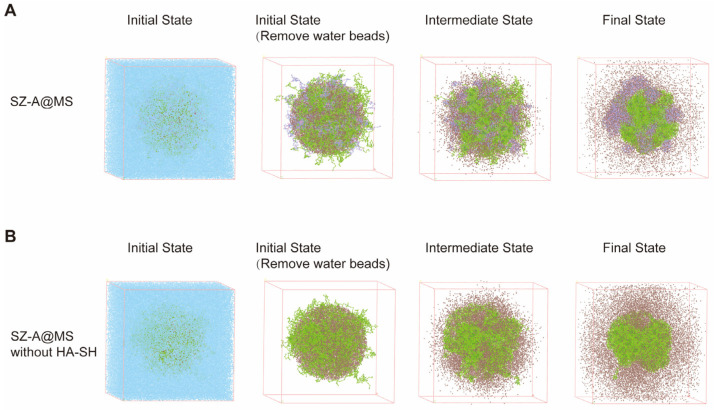
Simulation of SZ-A diffusion in microsphere systems using Materials Studio. (**A**) Simulated preparation of SZ-A@MSs. (**B**) Simulated preparation of SZ-A@MSs without HA-SH incorporation. Blue beads, brown beads, green beads, and purple beads represent water, SZ-A, PLGA, and HA-SH, respectively.

Dissipative Particle Dynamics (DPD) is a coarse-grained molecular simulation algorithm. The microsphere system was simulated by Materials Studio and the addition of HA-SH in the inner aqueous phase was modeled to affect the diffusion of SZ-A. Green beads represent PLGA, brown beads represent SZ-A, purple beads represent HA-SH, and blue beads represent water. The sphere is filled with PLGA and SZ-A, HA-SH is added according to the situation, and the rest is filled with water, and the entire system simulates microsphere preparation (Figure 4). The results show that the addition of HA-SH can reduce the escape of SZ-A from the inner aqueous core, indicating that the hydrogen bonding interaction between HA-SH and SZ-A can prevent SZ-A from leaking and diffusing into the outer aqueous phase during preparation, which is consistent with the experimental finding that HA-SH can increase the encapsulation rate of SZ-A in microspheres.

### 2.3. In Vitro Antioxidant and Anti-Inflammatory Effects of SZ-A@MS

**Figure 5:**
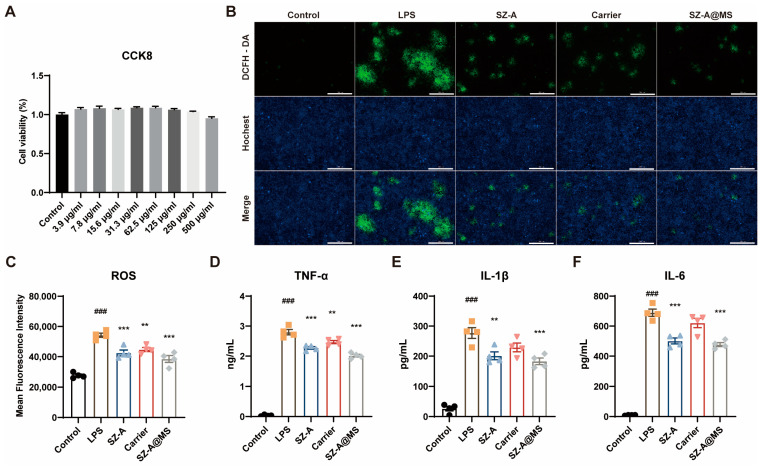
(**A**) Cell viability of Raw264.7 cells as determined by CCK-8 assay. (**B**,**C**) Intracellular ROS production and its statistical results (scale bar = 200 μm). Secretion levels of inflammatory factors (**D**) TNF-α, (**E**) IL-1β, (**F**) IL-6 in cell supernatants. Data are presented as the mean ± SEM. Significance: ^**^ *p* < 0.01, ^***^ *p* < 0.001 compared to LPS; ^###^ *p* < 0.001 compared to control.

The result of SZ-A@MSs affecting Raw264.7 cell viability, determined by the CCK8 method, is shown in Figure 5A. The results showed that compared with the normal group, the viability of Raw264.7 cells was greater than 95% for SZ-A@MSs in the dose range of 3.9–500 μg/kg. The drug-loaded microspheres had no inhibitory effect on the proliferation of Raw264.7 cells.

LPS is the main component of the membrane from Gram-negative bacteria [31]. It induces macrophages to polarize into a pro-inflammatory M1 phenotype, characterized by a significant increase in the secretion of IL-6, TNF-α, and IL-1β while triggering abnormal ROS accumulation through multiple mechanisms to form a characteristic inflammatory cascade amplification effect [32]. Specifically, ROS, byproducts of cellular metabolism predominantly generated in mitochondria, are overproduced via LPS-mediated mitochondrial dysfunction and NADPH oxidase activation [33]. This excessive ROS induce oxidative stress by damaging cellular components, which synergistically interact with inflammatory cytokine release to establish a self-perpetuating inflammatory feedback loop [34]. The LPS-triggered ROS/inflammatory cytokine network constitutes a critical pathological basis for sustained inflammatory progression, which can well simulate the inflammatory state of UC [35]. RAW264.7 cells are a mouse monocyte macrophage leukemia cell line whose functional characteristics are highly similar to activated peritoneal macrophages and thus is often used as a macrophage substitute model.

LPS-induced RAW264.7 cells were treated with SZ-A, carrier, and SZ-A@MSs. The cell supernatant was collected and the levels of the inflammatory factors IL-6, TNF-α, and IL-1β were determined using ELISA kits, and the intracellular oxidative stress levels were determined using ROS kits. The results showed that compared with the model group, the SZ-A@MS group had reduced ROS (Figure 5B,C) and reduced levels of secreted IL-6, TNF-α, and IL-1β (Figure 5D,F), indicating that the SZ-A@MSs have good anti-inflammatory and anti-ROS production effects. It is speculated that the anti-inflammatory effect of SZ-A@MSs is due to SZ-A and HA-SH, which have definite anti-inflammatory effects. The carrier group had certain anti-inflammatory and antioxidant effects, probably due to the presence of HA-SH. Hyaluronic acid has been shown to have good anti-inflammatory properties [36]. Combined with the results of CCK-8, the cell survival rate of each group was maintained above 95%, indicating that SZ-A@MSs do not exert their anti-inflammatory effect by inhibiting cell proliferation and killing cells.

### 2.4. Bio-Safety Assessment

**Figure 6:**
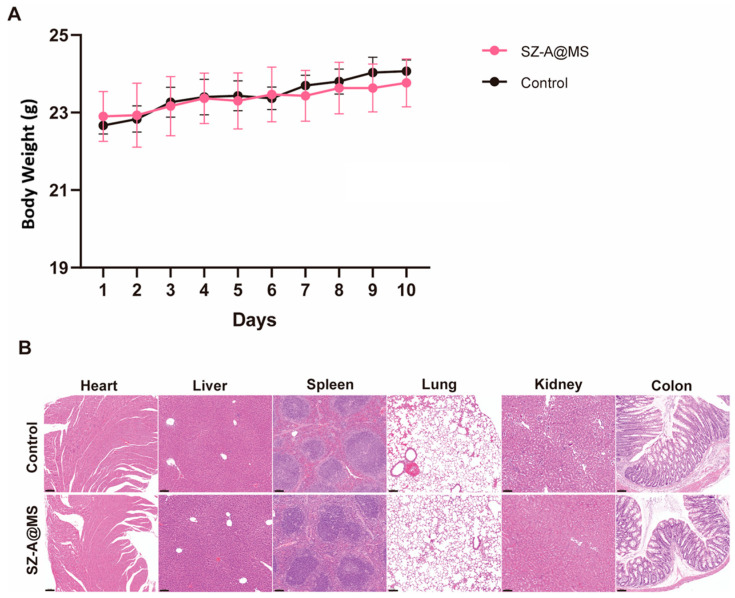
Results of microsphere safety evaluation. (**A**) Body weight. (**B**) H&E-stained histological sections of heart, liver, spleen, lung, kidney, and colon (scale bar = 100 μm). Data are presented as the mean ± SEM.

Normal mice were continuously administered SZ-A@MSs to evaluate the biosafety of the microspheres. Oral administration of drug-loaded microspheres for 10 consecutive days did not cause weight loss in mice compared to the control group (Figure 6A). In addition, H&E staining of heart, lung, colon, spleen, and kidney tissues of mice in each group showed that no significant pathological damage occurred in the tissues of each organ in the treated group (Figure 6B). These data indicate that SZ-A@MSs have good biocompatibility and a favorable safety profile.

### 2.5. Protective Effects of SZ-A@MSs on DSS-Induced Colitis

**Figure 7:**
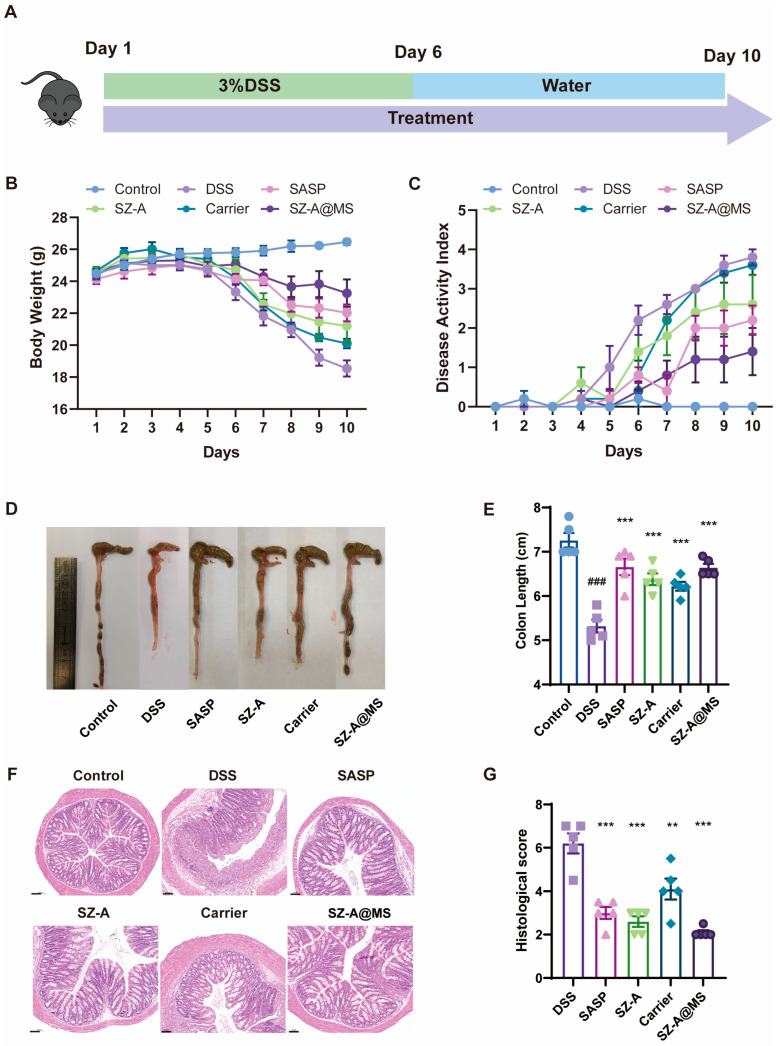
SZ-A relieved DSS-induced colitis in mice. (**A**) Schematic diagram of a mouse acute colitis model. (**B**) Body weight. (**C**) DAI score. (**D**) Pictures of colonic morphology in each experimental group. (**E**) Colon length. (**F**) H&E-stained histological sections of the colon of the mice (scale bar = 100 μm). (**G**) Histological scores. Data are presented as the mean ± SEM. Significance: ^**^ *p* < 0.01, ^***^ *p* < 0.001 compared to DSS; ^###^ *p* < 0.001 compared to control.

The mouse model of colitis was successfully constructed by allowing the mice to freely drink 3% DSS solution (Figure 7A). From the fifth day, the body weight of the mice in the model group began to decrease significantly (Figure 7B), and symptoms of diarrhea and bloody stools appeared, with the DAI score increasing (Figure 7C). After gavage with SZ-A@MSs, the trend of body weight loss slowed down, and the DAI score was significantly lower than that of the model group. The mice were dissected on the last day of the experiment, and the colon length of the mice was measured. The colon of the model group mice was atrophic, and the colon length was significantly reduced (Figure 7D,E). The colon length of the SZ-A@MS treatment group mice was increased compared with the model group. In addition, SZ-A@MSs showed better efficacy than SZ-A at the same dose. This may be attributed to the sustained-release properties of the microsphere formulation in the colon by targeting the colon locally, thereby prolonging the drug action time and improving bioavailability and reducing the amount of SZ-A released in the small intestine, thereby reducing the side effects of gastrointestinal flatulence caused by SZ-A in the small intestine. The middle section of the colon was selected for H&E staining. The results showed that the colon tissues of mice in the model group exhibited edema, cell necrosis, and disruption of structural integrity, accompanied by inflammatory infiltration (Figure 7F,G). The carrier group showed some improvement but still had tissue damage. The SZ-A group, SASP group, and SZ-A@MS treatment group could significantly improve the pathological changes in the colon, and the SZ-A@MS treatment group was superior. The active ingredient SZ-A plays a leading role in anti-inflammation and colon tissue repair, while the microsphere carrier itself can support SZ-A in anti-inflammation and further enhance the therapeutic effect by optimizing drug delivery.

**Figure 8:**
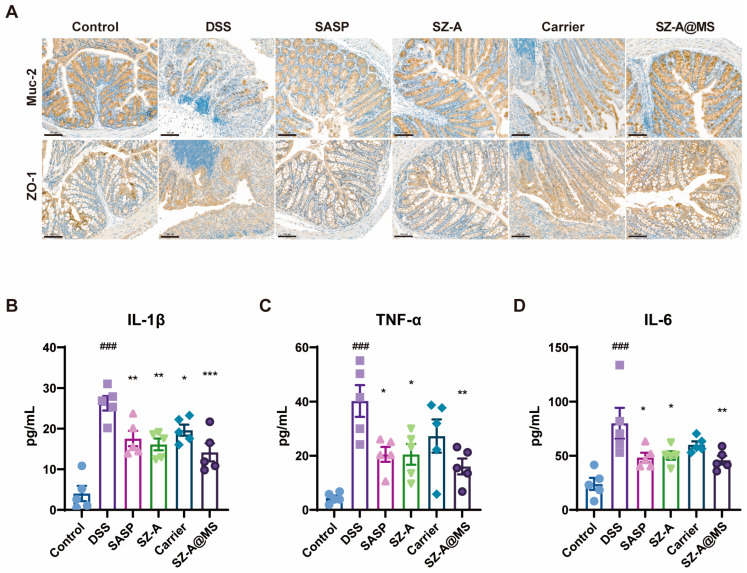
Expression of (**A**) Muc-2 and ZO-1 in the colon of the mice in each group determined by immunohistochemistry (scale bar = 100 μm). Serum levels of (**B**) IL-1β, (**C**) TNF-α, (**D**) IL-6 in each group of mice were determined by ELISA. Data are presented as the mean ± SEM. Significance: ^*^ *p* < 0.05, ^**^ *p* < 0.01, ^***^ *p* < 0.001 compared to DSS; ^###^ *p* < 0.001 compared to control.

Structural intestinal epithelial barrier dysfunction is a typical feature of UC. The intestinal barrier is an important physiological barrier that prevents harmful substances in the intestinal lumen, including bacteria, toxins, and antigens, from entering the body and is mainly composed of a chemical barrier in the mucus layer, a mechanical barrier in the epithelial cell layer, and an immune barrier in the lamina propria [37]. The mucus layer is composed of mucins secreted by goblet cells that form a protective gel covering the surface of intestinal epithelial cells. The main component is mucin 2, which can prevent microbial pathogens from directly contacting intestinal epithelial cells [38] while regulating intestinal permeability and the composition of the gut microbiota [39]. The epithelial cell layer consists of a single layer of columnar epithelial cells and their tight junctions. These intercellular tight junctions can selectively transport substances and prevent harmful substances such as bacteria and macromolecular inflammatory mediators in the intestinal lumen from passing through [40]. In this study, we investigated changes in the expression of key colonic barrier proteins by immunohistochemistry. In the intestinal barrier function, Muc-2 secreted by goblet cells is the main component of the mucus layer. ZO-1 is a tight junction protein that is expressed between the epithelial cells and can be in the formation of a selective permeability barrier. The experimental results showed that the DSS-induced colitis model group showed significant pathological changes compared to the control group (Figure 8A). The expression levels of ZO-1 and Muc-2 were significantly decreased in the DSS model group, presenting typical characteristics of barrier destruction. This synergistic downregulation of both proteins resulted in a double disruption of the intestinal physical and chemical barriers. The SZ-A@MS treatment group had a significant restoration effect, with a significant improvement in the pathological changes described above. The recovery of ZO-1 may contribute to the reconstruction of the selective permeability barrier between epithelial cells [41], while the upregulation of Muc-2 may resist pathogen invasion by enhancing the thickness of the mucus layer [42]. The synergistic effect of the two provides a double guarantee for the recovery of intestinal barrier homeostasis [43]. This suggests that SZ-A@MSs can ameliorate the disruption of the colonic barrier in mice.

UC leads to an elevated inflammatory response [44]. Cytokines are important signaling molecules in the immune system and play a key regulatory role in the initiation, maintenance, and extinction of the inflammatory response [45]. In UC, dysregulation of cytokine networks is one of the important characteristics of the disease pathophysiology [46]. Overexpression of pro-inflammatory cytokines leads to a sustained inflammatory response and tissue damage [47]. The inflammatory factors in the serum of mice were measured by ELISA, and the results showed that the pro-inflammatory factors of the DSS-induced model group mice were significantly increased, including TNF-α, IL-1β, IL-6 (Figure 8B–D). After treatment, the levels of inflammatory factors in each treatment group decreased, and the ability of the SZ-A@MS group to reduce inflammation was better than that of the carrier group. This result suggests that, combined with its effect of improving intestinal barrier function, SZ-A@MSs may exert a synergistic therapeutic effect by blocking the vicious cycle of inflammation and barrier disruption.

## 3. Materials and Methods

### 3.1. Materials

Sodium hyaluronate (400–1000 kDa) was obtained from Bloomage Biotechnology Corporation Limited (Jinan, China). Sodium alginate was obtained from Sigma-Aldrich (St. Louis, MO, USA). PLGA (75:25, 10–20 kDa) was from Yuanye Bio-Technology Co., Ltd. (Shanghai, China). SZ-A powder (lot number: J202309014) was kindly provided by Wehand-Bio Pharmaceutical Co., Ltd. (Beijing, China). Salazosulfapyridine (SASP) was purchased from SPH Sine Pharmaceutical Laboratories Co., Ltd. (Shanghai, China).

### 3.2. Preparation of the HA-SH

HA-SH was prepared according to previously reported methods [48]. Briefly, 24.0 mg of 3,3′-Dithiobis (propanoic dihydrazide), 111.0 mg of dimethoxy-1,3,5-triazin-2-yl-4-methyl morpholinium chloride solution (DMTMM), and 400.0 mg of HA were added in 50 mL MES buffer. The resulting solution was stirred at room temperature for 12 h. Then, 143.0 mg of tris(2-carboxyethyl) phosphine hydrochloride (TCEP-HCl) was added to the above gel system and stirred for 3 h to obtain a homogeneous solution. Finally, 1.0 g of NaCl was added, and the resulting HA-SH solution was purified by dialysis for 2 days followed by lyophilization. The material structure was determined by ^1^H NMR.

### 3.3. Characterization of Materials

Briefly, 100 mg/kg SZ-A and 4% HA-SH were mixed and allowed to form a hydrogel. The samples were determined after lyophilization by Fourier transform infrared (FTIR) spectrometry (Nicolet6700, Thermo Fisher Scientific, Waltham, MA, USA), set to scan over the wavelength range 4000–400 cm^−1^.

### 3.4. Preparation of the SZ-A@MS

Briefly, 4 mg HA-SH was dissolved in 200 µL 500 mg/mL SZ-A solution and mixed evenly as the inner aqueous phase W1. W1 was added to 2 mL dichloromethane solution (O) containing 100 mg/mL PLGA and emulsified using probe ultrasound (150 W, 2 mm ultrasound probe) to form a primary emulsion for 2 min. Then, 1% PVA and 1% sodium alginate were dissolved in 30 mL water to form the outer aqueous phase W2, and the primary emulsion was stirred and dispersed into W2 at 4500 rpm for 3 min. The primary emulsion was dispersed in W2 by stirring at 4500 rpm for 3 min. The resulting microsphere emulsion was stirred continuously for 3 h, then centrifuged at 4000 rpm for 5 min, washed twice with water, centrifuged, and lyophilized.

### 3.5. Characterization of SZ-A@MSs

Lyophilized microspheres were measured for particle size using a Mastersizer 2000 laser particle size analyzer (Malvern Panalytical, Malvern, UK). Particle size measurement range: 100 nm–2000 μm. The surface morphology of lyophilized microsphere powder was observed and photographed using a scanning electron microscope (SU8020, Hitachi High-Technologies, Tokyo, Japan). The porosity of the microspheres was determined by ImageJ (version 2.35).

A suitable amount of drug-loaded microspheres was weighed and ultrasonically dissolved, then the solution was filtered through a 0.22 μm filter membrane to obtain a clear liquid. The SZ-A content was determined using HPLC (Agilent Technologies, Santa Clara, CA, USA), and the drug loading and encapsulation efficiency of the microspheres were calculated.

Drug loading (DL) = (measured drug content/weight of microspheres) × 100%.

Entrapment efficiency (EE) = (measured drug content/actual drug added) × 100%.

### 3.6. In Vitro Release Profile of SZ-A@MSs

Lyophilized microspheres were placed in a dialysis bag and immersed sequentially in simulated gastric fluid (pH = 1.2) for 1 h, simulated intestinal fluid (pH = 6.8) for 2 h, and simulated colonic fluid (pH = 7.8) for 21 h. The microspheres were incubated on a constant temperature shaker at 37 °C. Dialysate samples were taken at specific time intervals. The SZ-A content in the dialysate samples was determined by high-performance liquid chromatography; the drug content was calculated and the cumulative drug release rate of the SZ-A@MSs was calculated using the following formula:

Cumulative drug release = Q_t_/Q_∞_.

Q_t_: cumulative drug release at time t.

Q_∞_: Theoretical maximum drug release.

### 3.7. In Vivo IVIS Imaging

The DSS-induced acute colitis model mice were subjected to a 24 h fast prior to imaging. The same fluorescence content of free IR820 fluorescent solution and IR820 fluorescent microspheres was administered orally. The mice were imaged using an In Vivo Imaging System (IVIS) at the following time points: 0, 1, 2, 4, 8, 12, 18, and 24 h. The resulting data were analyzed using LivingImage (version 4.4).

### 3.8. Ex Vivo Adhesion Experiments

Approximately 1 cm of mid-section colon tissue was obtained from healthy mice and acute colitis model mice. The intestinal contents were then removed by washing the tissues with physiological saline. The colon tissues were then placed in a PBS solution containing IR820 microspheres, after which they were shaken and incubated at 37 °C and 50 rpm for 30 min. The solution was then discarded, and the colon tissues washed three times with PBS. The colon tissues were then imaged under IVIS, and the fluorescence intensity was quantified using LivingImage (version 4.4).

### 3.9. Cell Cytotoxicity Assessment

Cells were cultured in 96-well plates with 6 replicate wells in each group and the number of cells in each well was adjusted to 1 × 10^5^. SZ-A@MS concentrations were set at 3.9, 7.8, 15.6, 31.3, 62.5, 125, 250, and 500 μg/mL. After 24 h of culture, cell viability was assessed by CCK-8 kit (Dojindo Laboratories, Kumamoto, Japan).

### 3.10. In Vitro Antioxidant and Anti-Inflammatory Evaluation

Twelve-well plates were used for cell culture, with 5 × 10^5^ RAW264.7 cells per well. They were divided into 6 groups: control group, LPS group (1 μg/mL LPS group), SZ-A group (1 μg/mL LPS+ 5 μg/mL SZ-A), carrier group (1 μg/mL LPS+ carrier), SZ-A@MS group (1 μg/mL LPS+ 40 μg/mL SZ-A@MSs). After culturing with 1 μg/mL LPS for 24 h, each drug was added and cultured for 24 h. Supernatants were collected for the determination of TNF-α, IL-6, and IL-1β levels by ELISA, and intracellular reactive oxygen species (ROS) were measured using the ROS assay kit.

### 3.11. Mesoscopic Simulations of SZ-A@MSs Based on Dissipative Particle Dynamics (DPD)

The key molecules in the system are coarse-grained. Intermolecular interaction parameters between beads are obtained using the Blends module in Materials Studio, and a DPD force field is created. The Build mesostructured template simulation is carried out in a system with dimensions of 100 × 100 × 100, where a core with a radius of 20 represents the initial state of the inner aqueous core. SZ-A, PLGA, and HA-SH beads are added to the core, and the remaining part of the system is filled with water beads to simulate the diffusion system. In DPD simulations, the coarse-grained structure of each molecule is constructed, and materials are added according to the prescription weight ratio at a temperature of 298 K (25 °C), with a total simulation length of 10,000 steps.

### 3.12. Animals and Experimental Design

The experimental animals were obtained from Beijing Huafukang Biotechnology Co., Ltd. (Beijing, China). Animals were maintained with free access to water and food. All animal experiments were conducted in accordance with the protocol approved by the Laboratory Animal Welfare and Ethics Committee of the Institute of Material Medicine, Chinese Academy of Medical Sciences (reference no.:00001706).

All C57BL/6J male mice were randomized into two groups. Mice in the control group received saline (0.9% NaCl) and mice in the SZ-A@MS group received SZ-A@MSs for 10 days (n = 3). Body weight changes were recorded and the mice were sacrificed on day 11. Heart, liver, spleen, lung, kidney, and colon tissues from each group were fixed in 4% paraformaldehyde and examined histopathologically by H&E staining.

Thirty male C57BL/6J mice (6–8 weeks old) were acclimatized for 1 week. The mice were randomly divided into six groups: control group, DSS group, SASP group (positive drug 300 mg/kg SASP treatment), SZ-A group (25 mg/kg SZ-A treatment), carrier group (microspheres without SZ-A), and SZ-A@MS group (equal to 25 mg/kg SZ-A). Except for the control group, mice were administered 3% DSS (M.W: 36,000–50,000; MP Biomedicals, Santa Ana, CA, USA) solution dissolved in freely available drinking water for 5 days. SZ-A or SASP was orally administered daily from the beginning to the end of the experiment. Samples were collected on day 10.

### 3.13. Disease Activity Index (DAI)

During the experiment, the body weights and fecal conditions of the mice were recorded daily. These measurements were used to calculate DAI as follows: DAI = weight loss score + fecal consistency score + bloody feces score. The severity of the different parameters was scored as follows: weight loss (no significant weight loss, 0; 1–5% loss, 1; 6–10% loss, 2; 11–20% loss, 3; >20% loss, 4), fecal consistency (normal stool, 0; consistent feces, 1; diarrhea, 2), and bloody feces (no bleeding, 0; fecal occult blood positive, 1; blood in feces, 2).

### 3.14. H&E Staining and Immunohistochemistry Staining

Colon tissues were fixed in 4% paraformaldehyde and embedded in paraffin. Sections were stained with hematoxylin and eosin (H&E) and photographed under a microscope. The protein expression in mice colon tissues was determined by immunohistochemistry staining with anti-mouse Muc-2 (1:2000, 27675-1-AP, proteintech) or anti-mouse ZO-1 (1:800,21773-1-AP, proteintech) antibodies.

### 3.15. Histopathological Scoring

Scoring was performed for edema (none, 0; localized increased cell spacing 1–5%, 1; generalized increased cell spacing 6–10%, 2; increased follicular spacing 11–20%, 3; increased follicular spacing over 20%, 4), inflammation (5 inflammatory cells at high magnification, 0.5 points; 10 cells, 1 point; 15 cells, 1.5 points; 20 cells, 2 points; 25 cells, 2.5 points; 30 cells, 3 points; >35 cells, 4 points), necrosis (5 necrotic cells at high magnification, 1 point; 10 necrotic cells at high magnification, 2 points; focal necrosis + fusion necrosis, 3 points; extensive fusion necrosis, 4 points), and hemorrhage (none, 0; hemorrhage, 1).

### 3.16. Serum and Cell Culture Supernatant ELISA Analysis

Tumor necrosis factor-α (TNF-α), IL-1β, IL-6 in serum and cell culture supernatant were determined using mouse ELISA kits (Elabscience, Wuhan, China).

### 3.17. Statistical Analysis

Statistical analyses were conducted using GraphPad Prism (version 9.0). Data are presented as the mean ± SEM. A one-way analysis of variance or unpaired two-tailed Student’s *t*-test was employed to analyze datasets containing two or more groups, depending on the experiment. Differences were considered statistically significant at *p* < 0.05.

## 4. Conclusions

In this study, we successfully developed a novel oral microsphere, the SZ-A@MS, for alleviating UC in mice. The SZ-A@MS is a sodium alginate-modified PLGA microsphere loaded with SZ-A. This microsphere exhibited excellent performance, with characteristics of highly efficient encapsulation of water-soluble drugs, reduced side effects of SZ-A, and good colonic targeting via intestinal adhesion and targeting of inflammatory sites. SZ-A@MSs effectively inhibited LPS-induced inflammation and oxidative stress in Raw264.7 cells. Animal studies further confirmed the ability of SZ-A@MSs to alleviate colitis. In conclusion, our findings suggest that SZ-A@MSs may be an effective strategy for targeted drug delivery in UC, providing a promising strategy to improve the therapeutic efficacy of SZ-A in the treatment of UC.

## Data Availability

The original contributions presented in this study are included in the article.
